# Omega-3 supplementation on inflammatory markers in patients with chronic Chagas cardiomyopathy: a randomized clinical study

**DOI:** 10.1186/s12937-017-0259-0

**Published:** 2017-06-09

**Authors:** Paula Simplício da Silva, Mauro Felippe Felix Mediano, Gilberto Marcelo Sperandio da Silva, Patricia Dias de Brito, Claudia Santos de Aguiar Cardoso, Cristiane Fonseca de Almeida, Luiz Henrique Conde Sangenis, Roberta Olmo Pinheiro, Alejandro Marcel Hasslocher-Moreno, Pedro Emmanuel Alvarenga Americano do Brasil, Andrea Silvestre de Sousa

**Affiliations:** 1 Serviço de Nutrição, Instituto Nacional de Infectologia Evandro Chagas, Fundação Oswaldo Cruz, Av. Brasil 4365, Manguinhos, Rio de Janeiro, Brazil; 2 Laboratório de Pesquisa Clínica em Doença de Chagas, Instituto de Pesquisa Clínica Evandro Chagas, Fundação Oswaldo Cruz, Av. Brasil 4365, Manguinhos, Rio de Janeiro, Brazil; 3 Laboratório de Hanseníase, Instituto Oswaldo Cruz, Fundação Oswaldo Cruz, Av. Brasil 4365, Manguinhos, Rio de Janeiro, Brazil

**Keywords:** Chagas disease, Chagas cardiomyopathy, Omega-3, Cytokines, Lipid profile

## Abstract

**Background:**

Several studies have been focusing on the effect of omega-3 polyunsaturated fatty acids on modulation of inflammatory markers in several cardiopathies. Although immunoregulatory dysfunction has been associated to the chronic cardiac involvement in Chagas disease, there is no study examining the effects of omega-3 supplementation in these patients. We investigated the effects of omega-3 PUFAs on markers of inflammation and lipid profile in chronic Chagas cardiomyopathy patients.

**Methods:**

The present study was a single-center double-blind clinical trial including patients with chronic Chagas cardiomyopathy. Patients were randomly assigned to receive omega-3 PUFAs capsules (1.8g EPA and 1.2g DHA) or placebo (corn oil) during an 8-week period. Cytokines, fasting glucose, lipid, and anthropometric profiles were evaluated.

**Results:**

Forty-two patients (23 women and 19 men) were included in the study and there were only two losses to follow-up during the 8-week period. Most of sociodemographic and clinical characteristics were similar between the groups at baseline, except for the cytokines IL-1β, IL-6, IL-8, IL-10, IL-17α, and IFNγ. The omega-3 PUFAs group demonstrated greater improvements in serum triglycerides (−21.1 vs. −4.1; *p* = 0.05) and IL-10 levels (−10.6 vs. −35.7; *p* = 0.01) in comparison to controls after 8 weeks of intervention. No further differences were observed between groups.

**Conclusion:**

Omega-3 PUFAs supplementation may favorably affect lipid and inflammatory profile in chronic Chagas cardiomyopathy patients, demonstrated by a decrease in triglycerides and improvements on IL-10 concentration. Further studies examining the clinical effects of omega-3 fatty acids supplementation in chronic Chagas cardiomyopathy are necessary.

**Trial registration:**

NCT01863576.

## Background

Chagas disease is a neglected disease infection caused by the protozoan *Trypanosoma cruzi* [1], affecting approximately 6 million people worldwide, mostly in Latin America [2]. Over the last years, migratory movements have been increasing the number of cases reported in non-endemic countries such as North America and Europe [3], with the possibility of non-vectorial transmission in these countries.

Chronic Chagas cardiomyopathy (CCC) is an important clinical manifestation of Chagas disease, including heart failure, ventricular and atrial arrhythmias, atrioventricular blocks, thromboembolism, stroke and sudden death [4–6]. The degree of heart dysfunction is associated with a progressive inflammatory reaction, which is characterized by a myocarditis with multifocal mononuclear inflammatory infiltrates, a varied degree of myocardium fibrosis, a constant low-grade tissue parasitism, and a low or undetectable parasitemia [7–10].

Immunoregulatory dysfunction has been speculated as an important mechanism related to the progression of Chagas disease [11]. The unbalance between excessive pro-inflammatory cytokines and decreased anti-inflammatory cytokines produces a low-grade pro-inflammatory state that could be associated to the progression of disease and the increased morbidity and mortality rates among these patients [11, 12].

Several studies have been focusing on the effect of nutritional interventions on modulation of inflammatory markers, including omega-3 polyunsaturated fatty acids (PUFAs) [13–15]. A study including 133 patients with a diagnosis of non-ischemic dilated cardiomyopathy and left ventricular systolic dysfunction (defined as an ejection fraction ≤ 45%) showed that omega-3 PUFAs supplementation promoted improvements on inflammatory markers, cardiac function and functional capacity in comparison to placebo after 12 months of follow-up. In addition, the hospitalization rates were 6% in the omega-3 PUFAs and 30% in the placebo group, highlighting the important clinical benefits promoted by omega-3 PUFAs supplementation among patients with systolic dysfunction [14].

The anti-inflammatory properties of omega-3 PUFAs are associated with short and long-term cardioprotective effects [16, 17]. The GISSI-HF trial, the major study evaluating the long-term effects of omega-3 PUFAs in individuals with chronic heart failure (CHF), showed a significant decrease in all-cause mortality and a composite of cardiovascular mortality and cardiovascular hospitalization in omega-3 PUFAs group in comparison to controls [13]. In addition, another study including 250 CHF patients achieved important improvements on cardiac function among those who received omega-3 PUFAs supplementation after six months of follow-up [18].

In this context, although several studies have been showing beneficial effects of omega-3 PUFAs on inflammatory and biochemical markers in individuals with CHF, none of them included patients with CCC and the potential effects of omega-3 PUFAs in this population is still unknown. In this study, we sought to investigate the effects of omega-3 PUFAs on inflammatory markers and lipid profile in CCC patients.

## Methods

### Study design

The present study was a single-center double-blind clinical trial conducted at the Evandro Chagas National Institute of Infectious Disease (Rio de Janeiro, Brazil). The full description of the study protocol have been published previously [19].

Patients aging > 18 years, with a diagnosis of CCC on stages B, C or D according to the current Brazilian Chagas disease consensus were included in the study [20]. The exclusion criteria were diarrheal disease, inflammatory bowel syndrome, the use of anti-inflammatory drugs, pregnant and lactating women, vitamin mineral or omega-3 PUFAs supplementation during the previous 30 days before the beginning of study, and the presence of cardiomyopathies other than CCC. All patients received information about the goals and the procedures of the study and signed an informed consent. The research protocol was approved by the local Institutional Review Board (CAAE-0037.0.009.000-10) and was registered in the ClinicalTrials.gov website (NCT01863576).

### Intervention

Patients were randomly assigned in a blinded fashion in a 1:1 ratio using sequentially numbered opaque sealed envelopes into one of the two groups: 1) an intervention group that received omega-3 PUFAs capsules (1.8g eicosapentaenoic acid and 1.2gdocosahexaenoic acid); 2) control group that received placebo (corn oil). The randomization list was computer-generated with blocking in advance (4 or 6 patients). The single researcher responsible for placing participants on study had no participation in the process of randomization.

Participants were counseled to take five omega-3 or placebo capsules per day (three in the morning and two the evening) and were followed during an 8-week period. They were also advised to not change their usual dietary habits during the study. The capsules were dispensed at the beginning of the study and after 4 weeks. The returned capsules were checked at weeks 4 and 8 to evaluate compliance to treatment.

The blister packaging and gel capsules of omega-3 PUFAs and placebo were identical and identified by the lot number on the package (Relthy Laboratories Ltda; Brazilian Health Surveillance Agency registration: 6.2582.0022.001-1).

### Measurements

Sociodemographic (age, sex, race and schooling) and clinical characteristics (NYHA functional class, alcohol consumption, smoking, and drugs prescription) were assessed at baseline using the information contained in the medical records. Food consumption and anthropometric measurements were evaluated thrice (baseline, after 4 and 8 weeks) whereas biochemical markers and cytokines were evaluated twice during the study (baseline and after 8 weeks).

Food intake was estimated by a single-trained nutritionist using the mean of 24-h dietary recalls assessed in three different moments during the study (weeks 0, 4, and 8) [21]. The 24-h dietary recall is a structured interview intended to capture detailed information about all foods and beverages consumed by the respondent in the past 24 h from midnight to midnight of the previous day. The dietitians entered dietary data directly into a nutrition analysis software (Diet Win plus 3.0® software package, Brubins Ltda.) to obtain the amount of carbohydrates, proteins, lipids, fiber, cholesterol, fatty acids (monounsaturated, polyunsaturated, saturated) and total calories intake.

The anthropometric evaluation consisted of measurements of height, body weight, waist and hip circumferences, tricipital skinfold thickness and arm circumference. Height and body weight were assessed with minimal clothes and without shoes using a calibrated digital scale coupled with a stadiometer. The body mass index (BMI) was calculated to determine the nutritional status [22]. Waist and hip circumferences were taken at the smallest girth of the waist and largest girth of the hip, respectively. An inelastic measuring tape was used to measure the mid-upper arm circumference and a scientific calibrated caliper (Cescorf®) was used to measure triceps skinfold thickness. The results were compared to the standards values established according to age [23, 24].

Blood samples were collected after 12h of overnight fasting. Aliquots of plasma and serum were isolated from the blood samples and frozen at −70 C within 2 h of being drawn. Total cholesterol, triglycerides, HDL-cholesterol, and glucose were measured using Siemens Dimension® reagent cartridge with an intra and inter-assay coefficients of variability <5%. The LDL- and VLDL-cholesterol concentrations were calculated according to the Friedewald equation based on the triglycerides measures [25]. Cytokine serum levels (IL-1β, IL-4, IL-6, IL-8, IL-10, IL-17-α, IL-33 TNF-α and IFN-γ) were measured in the serum of patients using specific sandwich enzyme-linked immunosorbent assays. Capture and detection antibodies were obtained from eBioscience kits (San Diego, CA, USA). The tests were conducted according to the manufacturer’s instructions.

### Blinding

All sociodemographic, clinical characteristics, food consumption and anthropometric measurements, biochemical markers and cytokines measurements were performed in a blinded fashion by the same single evaluator.

### Sample size

Considering a prevalence of 60% of inadequate omega-3 PUFAs intake [26], IFNγ values of 3986 (738) pg/mL at baseline and IFNγ values of 2922 (1275) pg/mL after omega-3 PUFA supplementation [27], and assuming 80% of power and 5% of significance level, the minimum estimated sample size was 40 patients (20 in each group).

### Data analysis

Baseline characteristics of the two groups were expressed as the mean (standard deviation) for continuous and percentages for categorical variables. Skewness and Kurtosis test was performed to evaluate the normality of data that were log-transformed in case of skewed distribution. Longitudinal changes between groups for all variables were examined using linear mixed models, which considers the treatment assignment and includes all observations of each participant regardless of loss to follow-up or compliance, characterizing an intention-to-treat analysis. The model included intervention group, time and time X intervention group interaction. The term of interest was time X group interaction, which estimates the rate of changes in the outcomes. The longitudinal model was adjusted for baseline values of the dependent variable in case of major unbalance for baseline values. Residual plots of all models were examined and did not show major deviations from regression assumptions. All statistical analysis were accomplished using Stata 13.0 software (College Station, TX, 2013) and significance level was set at *p* ≤0.05 for all analyses.

## Results

A total of 42 patients (23 women and 19 men) were included in the study from May, 2013 to September, 2013. Baseline characteristics of patients randomized to the two groups are shown in Table 1. Most of sociodemographic and clinical characteristics were similar between the groups at baseline, except for the cytokines IL-1β, IL-6, IL-8, IL-10, IL-17α, and IFNγ.

**Table 1:** Baseline characteristics of patients included in the study (*n* = 42)

Variable	Control (*n* = 21)	Intervention (*n* = 21)
Age (years)	55.0 (9.5)	58.6 (11.0)
Sex		
Male	38.1% (*n* = 08)	52.4% (*n* = 11)
Female	61.9% (*n* = 13)	47.6% (*n* = 10)
Income (Reais, R$)	1028.2 (556.9)	1168.9 (602.8)
Schooling		
Illiterate	14.3% (*n* = 3)	19.0% (*n* = 4)
Incomplete elementary school	61.9% (*n* = 13)	61.9% (*n* = 13)
Complete elementary school	14.3% (*n* = 03)	4.8% (*n* = 01)
Incomplete high school	4.8% (*n* = 01)	9.5% (*n* = 02)
High school	4.8% (*n* = 01)	4.8% (*n* = 01)
Ejection fraction (%)	45.7(14.8)	44.2(14.0)
Weight (kg)	67.3 (11.3)	69.1 (14.1)
Height (m)	1.56 (0.1)	1.60 (0.1)
Body mass index (kg/m^2^)	27.7 (5.2)	27.0 (4.7)
Brachial circumference (cm)	29.4 (4.2)	28.3 (3.9)
Triceps skinfold thickness (mm)	18.4 (11.1)	15.1 (7.7)
Brachial muscle circumference (cm)	23.3 (2.6)	23.7 (2.8)
Waist circumference (cm)	87.0 (9.6)	89.0 (12.8)
Hip circumference (cm)	100.4 (10.0)	99.5 (10.9)
Triacylglycerol (mg/dL)	93.8 (39.5)	97.7 (50.9)
Total cholesterol (mg/dL)	166.7 (27.5)	171.7 (38.8)
HDL-cholesterol (mg/dL)	50.4 (13.4)	51.1 (16.2)
LDL-cholesterol (mg/dL)	97.5 (23.2)	105.8 (32.9)
VLDL-cholesterol (mg/dL)	18.8 (8.0)	18.8 (9.1)
Glucose (mg/dL)	93.8 (9.5)	95.6 (8.0)
Tumour necrosis factor-α	66.4 (20.9)	65.6 (19.1)
Interleukin-4	22.2 (6.6)	23.1 (9.2)
Interleukin-6	335.4 (486.9)	163.9 (142.1)
Interleukin-8	84.8 (58.1)	59.8 (26.2)
Interleukin-10	105.2 (71.2)	70.4 (28.4)
Interleukin-33	77.2 (23.6)	86.7 (66.5)
Interleukin-1b	21.1 (8.9)	18.9 (4.4)
Interleukin-17a	5082.3 (5086.2)	3940.4 (4685.9)
Interferon-γ	75.5 (112.9)	47.8 (84.2)

Data are expressed as means (standard deviation) and prevalence of frequency

The majority of patients were in use of beta-blockers (69.1%) and 85% were in use of angiotensin-converting enzyme inhibitors/angiotensin II receptor blockers with no major treatment differences between groups (81%, *n* = 17 for control and 90.5%, *n* = 19 for intervention), except the omega-3 PUFAs intervention.

**Fig. 1:**
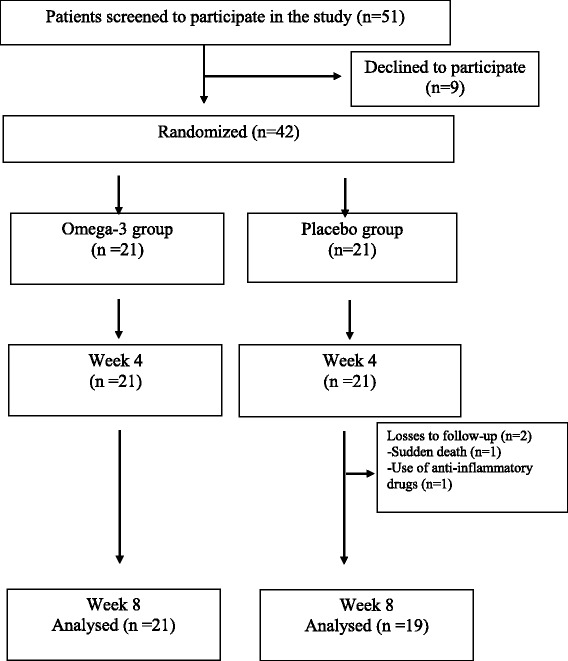
Progress of participants through the trial

Losses to follow-up during the 8-week period were 5% (one sudden death during a recreational soccer game and one patient in use of anti-inflammatory drug during the study period, both not related to the omega-3 PUFAs intervention) and the flow chart of patients included in the study is depicted in Fig. 1. Compliance levels were high for both groups, with no statistical differences to the percentage of ingested capsules at the end of follow-up (92.6% for placebo vs. 94.4% for omega-3 PUFAs; *p* = 0.41).

Crude means and changes from baseline for anthropometric, biochemical, and inflammatory variables are depicted in Tables 2, 3 and 4. No significant differences were found for carbohydrates, proteins, lipids, fiber, cholesterol, fatty acids (monounsaturated, polyunsaturated, saturated) and total calories intake between placebo and omega-3 PUFA groups during the entire follow-up period (Table 5). There were no differences between groups for any of the investigated anthropometric variables after 4 and 8 weeks of follow-up. However, the omega-3 PUFAs group demonstrated improvements in serum triglycerides (−21.1 vs. −4.1; *p* = 0.05) and IL-10 levels (−10.6 vs. −35.7; *p* = 0.01) in comparison to controls at the end of study (8 weeks). No further differences were observed between groups for biochemical and inflammatory markers. No adverse reactions associated with omega-3 PUFAs supplementation were reported during the follow-up.

**Table 2:** Crude means (standard deviation) and estimated changes from baseline (∆) for anthropometric variables during the follow-up

	4 week			8 week		
	(Control = 20; Intervention = 21)			(Control = 20; Intervention = 21)		
	Mean (DP)	∆^a^	*p* ^a^	Mean (DP)	∆^a^	*p* ^a^
Weight (kg)						
Control	67.9 (11.6)	+0.43		68.7 (10.2)	+0.52	
Intervention	68.2 (13.7)	+0.46	0.946	69.9 (13.4)	+0.84	0.386
Body mass index (kg/m^2^)						
Control	28.1 (5.2)	+0.18		28.4 (4.7)	+0.36	
Intervention	26.7 (4.7)	0.00	0.438	27.2 (4.5)	+0.21	0.542
Arm circumference (cm)						
Control	29.6 (5.0)	+0.04		29.9 (4.6)	+0.16	
Intervention	28.1 (4.2)	+0.03	0.986	28.6 (3.7)	+0.33	0.703
Tricipital skinfold thickness (mm)						
Control	18.4 (9.9)	−0.29		18.5 (10.1)	−0.17	
Intervention	15.7 (8.2)	+0.26	0.057	15.1 (7.6)	+0.14	0.261
*Arm circumference* (cm)						
Control	23.3 (2.3)	0.00		24.0 (2.8)	+0.48	
Intervention	23.6 (2.9)	+0.09	0.840	23.8 (2.7)	+0.08	0.323
Waist circumference (cm)						
Control	87.1 (9.5)	−0.28		88.1 (8.5)	+0.59	
Intervention	88.2 (12.7)	+0.20	0.263	89.1 (12.6)	+0.12	0.259
Hip circumference (cm)						
Control	100.3 (10.1)	−0.25		101.3 (9.1)	+0.11	
Intervention	99.0 (10.6)	+0.29	0.213	100.1 (10.1)	+0.59	0.258

^a^Linear mixed models include time, group and time x group interaction adjusted for baseline values

**Table 3:** Crude means (standard deviation) and estimated changes from baseline (∆) for biochemical variables

	8 week		
	(Control = 19 Intervention =21)		
	Mean (DP)	∆^a^	*p* ^a^
Triglycerides (mg/dL)			
Control	90.4 (35.3)	−4.1	
Intervention	76.6 (31.0)	−21.1	0.05
Total cholesterol (mg/dL)			
Control	168.5 (27.0)	+3.4	
Intervention	179.1 (34.6)	+7.4	0.60
HDL cholesterol (mg/dL)			
Control	49.7 (11.5)	−0.3	
Intervention	53.7 (15.1)	+2.6	0.31
LDL cholesterol (mg/dL)			
Control	100.7 (22.7)	+4.7	
Intervention	110.1 (31.0)	+4.3	0.96
VLDL cholesterol (mg/dL)			
Control	18.1 (7.1)	−0.9	
Intervention	15.3 (6.1)	−3.5	0.13
Glucose (mg/dL)			
Control	92.3 (13.3)	−2.0	
Intervention	95.2 (9.2)	−0.4	0.59

^a^Linear mixed models include time, treatment and time x treatment interaction, adjusted for baseline values

**Table 4:** Crude means (standard deviation) and estimated changes from baseline (∆) for inflammatory markers

	8 week		
	(Control = 19 Intervention =21)		
	Mean (DP)	∆^a^	*p* ^a^
Tumor necrosis factor-α (pg/dL)			
Control	84.5 (51.2)	+19.4	
Intervention	90.7 (48.8)	+27.2	0.58
Interleukin-4 (pg/dL)			
Control	21.5 (9.7)	−0.8	
Intervention	20.7 (6.7)	−1.5	0.67
Interleukin-6 (pg/dL)			
Control	67.3 (80.2)	−228.1	
Intervention	61.5 (54.3)	−85.3	0.45
Interleukin-8 (pg/dL)			
Control	64.7 (48.4)	−11.3	
Intervention	53.6 (12.4)	−3.3	0.47
Interleukin-10 (pg/dL)			
Control	53.8 (7.1)	−35.7	
Intervention	56.3 (8.3)	−10.6	0.01
Interleukin-33 (pg/dL)			
Control	70.4 (15.6)	−5.2	
Intervention	69.4 (17.0)	−5.5	0.97
Interleukin-1b (pg/dL)			
Control	18.0 (3.4)	−2.3	
Intervention	17.9 (3.4)	−0.6	0.24
Interleukin-17a (pg/dL)			
Control	3649.2 (4254.2)	+951.5	
Intervention	6130.0 (7644.2)	+3621.7	0.24
Interferon-γ (pg/dL)			
Control	82.6 (112.3)	+9.3	
Intervention	53.2 (100.7)	+7.1	0.90

^a^Linear mixed models include time, treatment and time x treatment interaction, adjusted for baseline values

**Table 5:** Means (standard deviation) of dietary variables and energy intake (*n* = 42)

Variable	Control (*n* = 21)	Intervention (*n* = 21)	*p*
Energy (kcal)	1487.59 (407.36)	1480.18 (419.08)	0.95
Carbohydrates (g)	202.75 (49.04)	200.59 (49.71)	0.89
Protein (g)	72.30 (27.77)	70.99 (26.00)	0.88
Total fat (g)	42.94 (19.93)	43.03 (19.50)	0.99
Fiber (g)	18.48 (7.01)	19.22 (7.75)	0.75
Cholesterol (mg)	215.60 (129.55)	205.70 (87.81)	0.77
Saturated fatty acids (g)	12.60 (5.93)	13.81 (7.62)	0.57
Monounsaturated fatty acids (g)	12.36 (7.82)	13.15 (8.02)	0.75
Polyunsaturated fatty acids (g)	9.54 (5.70)	8.41 (3.80)	0.45
Trans fatty acids (g)	0.65 (0.58)	0.80 (0.76)	0.48
Linoleic acid, n-6 (g)	6.17 (4.24)	5.65 (2.99)	0.65
Alfa-linoleic acid, n-3 (g)	0.72 (0.80)	0.56 (0.32)	0.42

## Discussion

Omega-3 PUFAs supplementation has been demonstrating significant effects on prevention and treatment of many chronic health conditions, including heart failure [13, 28, 29]. A prospective cohort study conducted by Mozzafarian et al.[30] demonstrated an important association between an increased omega-3 PUFAs concentration and a lower incidence of congestive heart failure (HR 0.70; CI 95% 0.49–0.99) following 2735 U.S. adults free of heart disease during 14 years.

The physiological effects of omega-3 PUFAs supplementation are associated to the incorporation of long chain omega-3 PUFAs into the cell membranes, modulating their biophysical properties and functionality. PUFAs are biosynthesis precursors of several important metabolites, especially eicosanoids (prostaglandins, leukotrienes, thromboxanes, lipoxins, and others), synthesized from arachidonic acid [31]. Omega-3 PUFAs reduce arachidonic acid content in cell membranes, resulting in the synthesis of eicosanoids that have greater anti-inflammatory properties than the eicosanoids derived from omega-6 PUFAs [32]. The American College of Cardiology and American Heart Association (ACC/AHA) considers that omega-3 PUFAs supplementation is an important adjuvant in the heart failure therapy [33].

To our knowledge, the present study was the first one to demonstrate a reduction of serum triglycerides as result of the omega-3 PUFAs supplementation in patients with CCC. This is important given that elevated triglyceride levels are considered independent predictors of cardiovascular risk [34]. Results from preclinical and clinical studies suggested that omega-3 PUFAs decrease serum triglycerides concentrations by reducing its synthesis, reducing the triglyceride incorporation into VLDL, reducing triglyceride secretion, and enhancing triglyceride clearance from VLDL particles [15]. In a study conducted by Kastelein et al. [35], 399 subjects with severe hypertriglyceridemia (≥500 mg/dL to <2000 mg/dL) were randomly assigned to receive placebo (olive oil 4 g/d) or omega-3 PUFAs supplementation at different dosages (2 g/d, 3 g/d or 4 g/d). Fasting serum triglycerides changed from baseline by −25.9% (*p* < 0.01 vs. placebo), –25.5% (*p* < 0.01 vs. placebo), and −30.9% (*p* < 0.001 vs. placebo) for 2g/d, 3g/d, and 4g/d of omega-3 PUFAs, respectively. In another study, patients with metabolic syndrome treated with 2 g/d of omega-3 PUFAs also found a decrease in triglycerides levels from day 0 to 28 and 84 (*p* < 0.01) and in serum total cholesterol levels (*p* < 0.01), highlighting the importance of omega-3 supplementation on improvements of lipid profile [36].

Most of studies involving omega-3 supplementation have been demonstrating a decreased production of pro-inflammatory cytokines such as TNF-α, IL-1β and IL-6 [37, 38]. Similar results were observed in a meta-analysis that evaluated of the effects of fish oil supplementation in patients with chronic heart failure, showing that circulating TNFα, IL-1β, and IL-6 decreased after 3 to 12 months of follow-up [39].

In our study, omega-3 PUFAs were able to attenuate the decrease observed in the IL-10 concentration (an anti-inflammatory cytokine) at the end of follow-up, showing an important positive effect of omega-3 PUFAs supplementation on inflammatory modulation in patients with CCC. Although these results have been demonstrated by studies conducted in several different populations [40–42], those examining the effects of omega-3 PUFAs supplementation on IL-10 in patients with HF presented controversial results. For instance, no changes for IL-10 were observed in a study including 16 male patients with HF that received omega-3 PUFAs supplementation during 60 days [43]. In addition, another study including 190 patients with paroxysmal or persistent atrial fibrillation showed no significant effects of omega-3 PUFAs supplementation on IL-10 concentration after a 6-month period [44].

Specific immune expression of anti-inflammatory and pro-inflammatory cytokines may play a relevant role in the clinical presentation of chronic Chagas disease [45]. *T. cruzi* infection induces a strong inflammatory response dominated by the Th1 pattern, with the IFN-γ and TNF-α expressions regulated by the IL-10 production [46]. The immunological balance in patients with indeterminate form has a high expression of IL-10, whereas cardiac form is associated with a reduction of IL-10 production and increased production of TNF-αand IFN-γ. The pro-inflammatory cytokines IL-12, IFN-γ and TNF-α activate macrophages to promote parasite killing through the production of trypanocidal radicals. These cytokines also acts as a positive feedback for Th1 differentiation. Th1 cells orchestrate an expressive CD8+ T cell response, causing tissue damage and fibrosis [46, 47]. Correlation analysis showed that higher IL-10 expression was associated with better cardiac function, as determined by left ventricular ejection fraction and left ventricular diastolic diameter values [12]. In this way, the results of our study demonstrate a positive influence of omega-3 PUFAs supplementation upon inflammatory profile in patients with CCC.

The present study has some limitations. Although sample size has been calculated to identify longitudinal statistical differences for cytokines between omega-3 PUFAs and placebo groups, this small number of patients may have contributed to the differences observed between groups for some cytokines at baseline. However, we included baseline values in longitudinal models in order to minimize the influence of baseline imbalance on statistical analysis. The short-term follow-up period did not allow us to evaluate clinical outcomes, though that was not the scope of the present study. In addition, the lack of a health control group and the absence of a physical activity measurement could make the interpretation of results difficult. Strengths included the very low frequency of losses to follow-up and the high compliance rates, which are very difficult to achieve in clinical trials [48].

To conclude, omega-3 PUFAs supplementation may favorably affect lipid and inflammatory profile in chronic Chagas cardiomyopathy patients, demonstrated by the decrease in triglycerides and improvements on IL-10 concentration. This results highlights to the importance of omega-3 PUFAs supplementation as a new coadjuvant strategy to treat patients with CCC, promoting a better control of inflammatory parameters and triglycerides. Studies evaluating the effects of increased consumption of omega-3 rich foods as well as the long-term effects of omega-3 PUFAs on inflammatory profile and clinical outcomes in CCC requires further investigation.

## Abbreviations

BMI: Body mass index
CCC: Chronic Chagas cardiomyopathy
CHF: Chronic heart failure
PUFAs: Polyunsaturated fatty acids
